# EGFR mRNA-Engineered Mesenchymal Stem Cells (MSCs) Demonstrate Radioresistance to Moderate Dose of Simulated Cosmic Radiation

**DOI:** 10.3390/cells14211719

**Published:** 2025-11-01

**Authors:** Fay Ghani, Peng Huang, Cuiping Zhang, Abba C. Zubair

**Affiliations:** Center for Regenerative Biotherapeutics and Department of Laboratory Medicine and Pathology, Mayo Clinic, Jacksonville, FL 32224, USA; abdulghani.fay@mayo.edu (F.G.); huang.peng@mayo.edu (P.H.); zhang.cuiping@mayo.edu (C.Z.)

**Keywords:** radiation, mRNA engineered MSCs, epidermal growth factor receptor, spaceflight

## Abstract

Galactic cosmic ray (GCR) radiation is a major barrier to human space exploration beyond Earth’s magnetic field protection. Mesenchymal stem cells (MSCs) are found in all organs and play a critical role in repair and regeneration of tissue. We engineered bone marrow-derived MSCs and evaluated their response to ionizing radiation exposure. Epidermal growth factor receptor (EGFR) expression by certain types of cancers has been shown to induce radioresistance. In this study, we tested the feasibility of transfecting MSCs to overexpress EGFR (eMSC-EGFR) and their capacity to tolerate and recover from X-ray exposure. Quantitative real-time PCR (qRT-PCR) and immunoblotting results confirmed the efficient transfection of EGFR into MSCs and EGFR protein production. eMSC-EGFR maintained characteristics of human MSCs as outlined by the International Society for Cell & Gene Therapy. Then, engineered MSCs were exposed to various dose rates of X-ray (1–20 Gy) to assess the potential radioprotective role of EGFR overexpression in MSCs. Post-irradiation analysis included evaluation of morphology, cell proliferation, viability, tumorigenic potential, and DNA damage. eMSC-EGFR showed signs of radioresistance compared to naïve MSCs when assessing relative proliferation one week following exposure to 1–8 Gy X-rays, and significantly lower DNA damage content 24 h after exposure to 4 Gy. We establish for the first time the efficient generation of EGFR overexpressing MSCs as a model for enhancing the human body to tolerate and recover from moderate dose radiation injury in long-term manned space travel.

## 1. Introduction

Deep space travel unlocks new adventures for humans to go further into the cosmos than ever before while also raising serious concerns about their health and safety in doing so. Participants in deep space missions face exposure to space radiation impacting a variety of body systems and physiological processes, including the ionization of tissues, DNA damage, and other detrimental effects. This is a result of the dynamic and complex high- and low-energy particles found in space, like galactic cosmic rays (GCRs), solar energetic particles (SEPs), and the radiation trapped by the Earth’s magnetic field [1,2,3,4]. With the advancements in human spaceflight technologies and the movement of humans beyond suborbital flight and low Earth orbit to lunar settlement and long-duration spaceflight, the effects of space radiation on human health need to be fully understood.

Mesenchymal stem cells (MSCs) are multipotent adult stem cells found in the bone marrow which play a critical role in the repair and regeneration of tissue. The exposure of MSCs to radiation during deep space travel and long-duration spaceflight could have negative effects on the role of MSCs in maintaining homeostasis and downstream effects. This includes the hematopoietic system, which is highly sensitive to ionizing radiation due to the rapid turnover and proliferation of cells [5]. MSCs play a vital role in supporting the hematopoietic system by forming the bone marrow stromal niche that regulates hematopoietic stem cell (HSC) maintenance, self-renewal and differentiation [6]. Epidermal growth factor receptor (EGFR) has been studied in many types of malignancies for its role in treatment radioresistance and radiation-induced EGFR signaling [7,8,9]. It has already been shown that MSCs exposed to simulated GCR/SEP radiation experience dramatic changes in their differentiation potential and induce DNA damage and mutations resulting in leukemic transformation within the hematopoietic system [10,11]. Better shielding and increases in the human body’s capacity to protect and heal itself after cosmic radiation exposure will increase the feasibility and safety of human space exploration.

In this study, a robust protocol for the mRNA transfection of human bone marrow-derived MSCs with EGFR mRNA was developed. Then, a subsequent assessment of how transfected cells respond to various doses of X-ray was performed, uncovering potential synergistic effects relevant to enhancing their radioresistance. Based on widely accepted radiobiological literature, single-dose exposures in the range of 0.1–1 Gy are considered relatively low while 1–5 Gy are referred to as moderate since they are high enough to induce measurable biological effects and below the threshold for immediate cell lethality, allowing for post-irradiation analysis. The dose range of 5–10 Gy is considered as high while ranges of 10–20 Gy are considered very high and can cause severe cellular damage [12,13,14]. Our study investigates the effects of moderate-to-very-high single-dose ionizing radiation classification on MSCs. First, a methodological establishment of EGFR mRNA transfection into MSCs was achieved (eMSC-EGFR), including an assessment of MSC functional characteristics. Then, a comprehensive analysis of eMSC-EGFR following irradiation was performed. Our study has demonstrated for the first time that it is feasible to efficiently generate EGFR overexpressing MSCs with enhanced radioresistance capacity following irradiation compared to naïve MSCs while also exhibiting normal MSC behavior. The novelty of this study lies in its investigation of how mRNA transfection alters radioresistance in MSCs to X-ray exposure, offering new insights into their behavior which may inform strategies for enhancing cell-based therapies in therapeutic and radiological contexts. We provide a new approach to protecting MSCs from the insults of radiation present during deep space travel, benefiting space travelers and the performance of biomedical experiments in deep space.

## 2. Materials and Methods

### 2.1. Synthesis of mRNA

The human EGFR open reading frame sequence (Homo sapiens EGFR, mRNA (NM_005228.3)) was obtained from National Center for Biotechnology Information website (https://www.ncbi.nlm.nih.gov/nuccore/NM_005228.3/, accessed on 10 January 2024). EGFR mRNA was obtained from TriLink BioTechnologies (San Diego, CA, USA) and contained T7 promoter, 5′ untranslated regions (UTRs), 3′ UTRs and poly A-tail in the sequence. CleanCap^®^ enhanced green fluorescent protein (EGFP) mRNA (TriLink BioTechnologies, Cat# L-7601) served as a control to confirm successful transfection.

### 2.2. Cell Culture and mRNA Transfection

Human bone marrow-derived mesenchymal stem cells (BMSCs) were isolated from commercial de-identified bone marrow from a healthy 22-year-old male donor (AllCells, Alameda, CA, USA). BMSCs were cultured in Minimum Essential Medium α (MEM α; Thermo Fisher Scientific (Gibco™), Carlsbad, CA, USA, Cat# 12561072) supplemented with 16.5% FBS, 1% Glutamax and 1% penicillin–streptomycin (Thermo Fisher Scientific, Grand Island, NY, USA) in a 37 °C, 5% CO_2_ humidified incubator. 0.05% Trypsin-EDTA (Gibco™, Cat# 25300120) was used to perform the subculture.

At passage three, MSCs were plated with a seeding density of 1.30 × 10^5^ cells/well (1.30 × 10^4^ cells/cm^2^) into six-well plates and cultured for at least 24 h until reaching 80–90% confluency. The transfection of MSCs was performed using 1.25 µg/mL EGFR mRNA or EGFP mRNA and 1.875 µL/mL Lipofectamine MessengerMAX Reagent (LMM; Thermo Fisher Scientific (Invitrogen^TM^), Carlsbad, CA, USA, Cat# LMRNA015) according to the manufacturer’s instructions. A time course experiment was performed where images were captured, mRNA was isolated, and conditioned culture medium (CCM) was collected at 4, 12, 24 and 48 h post-transfection along with 4 days and 7 days. Naïve MSCs were included which underwent the same procedure but without mRNA transfection. MSCs were harvested for analysis within passage 4 in the study.

### 2.3. Viability

Th viability of naïve and engineered MSCs was evaluated using the Nexcelom Cellometer Auto 2000 Cell Viability Counter (Revvity, Inc., Lawrence, MA, USA) and ViaStain^TM^ AOPI Staining Solution (Revvity, Inc.), following 24 h, 48 h, 4 days and 7 days of transfection. Viability was assessed again after irradiation of MSCs with X-ray at several timepoints from day 1 up to day 14.

### 2.4. MSC Phenotyping

MSC characterization was evaluated by flow cytometry using the MACSQuant Analyzer 16 (Miltenyi Biotec Inc., Auburn, CA, USA) and the MSC Phenotyping Kit (Miltenyi Biotec Inc., Cat# 130-125-285) to detect MSC positive markers (CD73, CD90, and CD105) and MSC negative markers (CD14, CD19, CD34, CD45, and human leukocyte antigen-DR isotype (HLA-DR)). Compensation was carried out before initiating every flow cytometry assay using the MACS^®^ Comp Bead Kit (Miltenyi Biotec Cat# 130-104-187). Gating strategy for flow cytometry analysis is included in Supplementary Figure S1.

### 2.5. Differentiation of MSCs

MSC differentiation potential was evaluated by culturing naïve and engineered MSCs in osteogenic and adipogenic differentiation assays. For adipogenic differentiation, MSCs were cultured using StemPro adipogenesis differentiation kit (Gibco^TM^, Cat# A1007001) and supplemented with 1% penicillin–streptomycin (Thermo Fisher Scientific). The medium used for differentiation was changed every 3–4 days until day 14. Cells were then fixed using 10% formalin for 1 h at room temperature and stained with oil-red-O working solution for 20 min at room temperature. The preparation involved combining three portions of 0.5% oil-red-O solution and two portions of Dulbecco’s phosphate-buffered saline. For osteogenic differentiation, StemPro osteogenesis differentiation kit (Gibco, Cat# A1007201) was used and supplemented with 1% penicillin–streptomycin. The differentiation medium was replaced every 3–4 days until Day 21. Cells were then fixed with 10% formalin and stained with alizarin red S staining. Adipogenic and osteogenic differentiation controls were included where MSCs were cultured in MEM α supplemented with 1% penicillin–streptomycin, 1% Glutamax, and 16.5% FBS and stained with oil-red-O working solution or alizarin red S solution, respectively. Images were acquired by bright field microscope.

### 2.6. Quantitative Reverse Transcription Polymerase Chain Reaction (qRT-PCR)

RNA was isolated from cultured cells using RNeasy Plus Mini Kit (QIAGEN Inc., Germantown, MD, USA, Cat# 74134) while reverse transcription was performed using QuantiNova Reverse Transcription Kit (QIAGEN Inc., Cat# 205411). The NanoDrop 2000 Spectrophotometer (Thermo Fisher Scientific, Wilmington, DE, USA) was used to measure RNA concentration. For real-time PCR, TaqMan Fast Advanced Master Mix (Applied Biosystems (Thermo Fisher Scientific), Carlsbad, CA, USA, Cat# 4444963), EGFR (Thermo Fisher Scientific, Hs01076090_m1, Cat# 4331182), and glyceraldehyde 3-phosphate dehydrogenase (GAPDH) (Hs02758991_g1), Gene Expression Taqman Assays were used. GAPDH was used as an internal control. The 2^−ΔΔCT^ method was used for the analysis of relative gene expression of EGFR. Samples were assessed in triplicate and the mean value was taken for analysis.

### 2.7. Western Blot Analysis

MSCs cultured in the six-well plate were collected for Western blot detection of EGFR protein 24 h post-transfection. BT-20, a human breast adenocarcinoma cell line originating from the tumor of a 74-year-old Caucasian female and known to express high levels of EGFR, was included in the study to serve as a reference for comparison with MSCs. BT-20 cells were cultured in Eagle’s Minimum Essential Medium (EMEM; ATCC (American Type Culture Collection), Manassas, VA, USA, Cat# 30-2003) supplemented with 10% FBS for 24 h prior to sample preparation for Western blot analysis. Cultured cells were washed once with ice cold phosphate-buffered saline (PBS; 0.1 M phosphate, 0.15 M sodium chloride; pH 7.2) and then scraped off the plate using a cell scraper and a lysis buffer composed of RIPA Lysis and Extraction Buffer (Thermo Scientific™, Cat# 89900) and cOmplete™, Mini Protease Inhibitor Cocktail (Sigma-Aldrich Inc., St Louis, MO, USA, Cat# 11836153001). The normalization of protein concentration was achieved using the Pierce BCA Assay (Thermo Fisher Scientific, Waltham, MA, USA). Protein lysates were denatured at 95 °C for 5 min. Lysates with normalized protein concentrations were loaded onto a NuPAGE 4–12% Bis-Tris Mini Protein Gel (Invitrogen, Thermo Fisher Scientific, Waltham, MA, USA) transferred to an Immobilon-P polyvinylidene fluoride membrane (MilliporeSigma, Burlington, MA, USA) via electrophoresis. The membrane was placed in blocking solution containing 5% non-fat dry milk in Tris-buffered saline with 0.1% Tween 20, and then incubated overnight at 4 °C with continuous agitation with the following primary antibodies: human anti-EGFR at 1:500 (Santa Cruz Biotechnology, Inc., Dallas, TX, USA, Cat# sc-373746) and anti-βactin at 1:1000. The next day, the membrane was washed with Tris-buffered saline with 0.1% Tween 20 three times for 10 min each wash and then incubated with horseradish peroxidase-conjugated secondary antibodies at a 1:2000 dilution for 1 h at room temperature while being rocked. Relative EGFR expression levels were normalized to the loading control βactin. Protein expression was detected via chemiluminescence. ImageJ 1.54d software was used to analyze the density of the immunoreactive bands where background was subtracted followed by normalization to the loading control obtained from the same gel (βactin) and a percentage relative to the control cells (naive MSCs) was calculated.

### 2.8. Cell Proliferation Assay

MSCs were seeded into a 96-well plate at a sending density of 2500 cells/well and cultured for 24 h in a humidified incubator. Cell proliferation was assessed using Cell Counting Kit-8 (CCK-8; Dojindo Laboratories, Kumamoto, Japan, Cat# CK04-13) by measuring spectrophotometric absorbance at 450 nm according to manufacturer’s instructions. Optical density measurements were recorded on Day 1, 2, 3, 4 and 5 following seeding of cells, with Day 1 being the baseline measurement taken following the 24 h incubation. Each group was assessed in triplicate.

### 2.9. Cell Irradiation (X-Ray)

Twenty-four hours post-transfection with EGFR, MSCs were collected and seeded onto 24-well plates (3 × 10^4^ cells/well) and left to attach in a humidified incubator. After an overnight culture, cell cultures were irradiated using the X-RAD 160 (Precision X-Ray Inc., North Branford, CT, USA), a cabinet-based X-ray irradiator designed for high-throughput and precise dose delivery. Samples were placed on a motorized rotating shelf at a fixed source-to-sample distance (SSD) of 33 cm. To ensure uniform dose delivery, plates were centered using the system’s laser alignment and real-time imaging system. Irradiation was performed at 160 kV and 18.70 mA, delivering doses ranging from 0 to 20 Gy, depending on the experimental condition. All irradiation was conducted at room temperature, and plates were returned to the incubator immediately after exposure. For DNA damage analysis, MSCs were seeded to µ-Slide 8 Well (ibidi GmbH, Gräfelfing, Bavaria, Germany) at 5 × 10^3^ cells/well and then irradiated.

### 2.10. Tumorigenicity Assay

The Soft agar assay was used to evaluate the tumorigenicity of naïve MSCs and transfected MSCs after 4 Gy (low dose) and 20 Gy (high dose) X-ray exposure as well as no exposure (0 Gy). Human fibrosarcoma cell line HT-1080 (ATCC, CCL-121) and fibroblast cell line WI-38 (ATCC, CCL-75) were used as positive and negative controls, respectively. Cells were suspended in 2 mL of top agar (0.35% agar in α-minimum essential medium containing 20% FBS) and then plated on 3 mL of bottom agar (0.5% agar in α-minimum essential medium containing 20% FBS) in a 6-well plate. Each sample was tested at two concentrations in triplicate: low concentration (5000 cells/well) and high concentration (67,000 cells/well). Colonies were identified after a 21-day incubation and images captured using light microscopy.

### 2.11. DNA Damage

Bone marrow-derived MSCs (5 × 10^3^ cells/well) were seeded to µ-Slide 8 Well (ibidi) and grown to ~90% confluency and then irradiated using X-RAD at 0 Gy (no radiation), 4 Gy, 8 Gy and 20 Gy. Cells were prepared for immunostaining 24 h post-irradiation. Positive control wells were stimulated with 100 µM etoposide (Abcam Inc., Waltham, MA, USA) for 2 h prior to staining to induce DNA damage. First, cells were washed three times with PBS and fixed with 4% PFA for 15 min at 37 °C. Following fixation, cells were washed three times with PBS and permeabilized with 0.1% Triton-X 100 in PBS for 4 min at room temperature. Samples were blocked with 3% bovine serum albumin and 0.05% Tween-20 in PBS for 1 h at room temperature to reduce non-specific binding. Primary antibody pS.139-H2A.X antibody (Cell Signaling Technology, Inc., Danvers, MA, USA) diluted in blocking buffer at 1:250 was applied to cells and incubated for 24 h at 4 °C. After five washes with PBS, fluorescently labeled goat anti-rabbit secondary antibody, Alexa Fluor 488 (Thermo Fisher Scientific) at 1:750 and nuclei stain 4′,6-diamidino-2-phenylindole (Sigma-Aldrich (Merck KGaA), St. Louis, MO, USA) at 1:5000 in blocking solution were added for 2 h at room temperature in the dark. Following this, samples were washed five times with PBS and mounting media (ibidi) was added to the wells. The EVOS FL Cell Imaging System (Thermo Fisher Scientific) was used to capture images while ImageJ software was used to conduct the quantification. Since analysis was conducted on the nuclei of the same cell type and assuming that the nuclei are similar in size and shape (the area is consistent across the regions of interest), the mean gray value was used to reflect the average fluorescence intensity per pixel, to compare relative DNA damage levels across conditions. The mean fluorescence intensity within each cell was measured followed by background subtraction, averaging, then normalization against the average measured from the non-irradiated cells (0 Gy) or etoposide-treated cells (positive control). Forty cells were counted for each sample (*n* = 40).

### 2.12. Statistical Analysis

GraphPad Prism 10 software (Boston, MA, USA) was used for statistical analysis. For groups with significantly different standard deviations as assessed by the Brown–Forsythe test, the Brown–Forsythe and Welch ANOVA tests with Dunnett T3 multiple comparisons test with individual variances computed for each comparison were performed. Ordinary one-way ANOVA with no pairing and matching with the assumption of Gaussian distribution of residuals was conducted for the mean GFP fluorescence intensity, EGFR relative RNA expression and EGFR immunoblotting analyses. An ordinary two-way ANOVA was conducted for cell proliferation and EGFR relative RNA expression analyses without assuming sphericity (equal variability of differences) and using the Geisser–Greenhouse correction. For DNA damage analysis, two-way ANOVA with Tukey’s post hoc test was conducted to assess differences between mean of etoposide-treated cells and non-treated cells, and between the means of naïve MSCs and eMSC-EGFR at each dose. Then, a comparison was made between naïve MSCs and eMSC-EGFR where each condition was compared to non-irradiated cells (normalized to 0 Gy) to allow for an easier comparison of how fluorescence changes relative to radiation exposure. Parametric *t*-test (unpaired, two-tailed) with Welch’s correction was used to compare naïve vs. engineered MSCs at each dose independently. Two-way ANOVA was performed for a post-irradiation viability assay to assess the effects of group (naïve MSCs and eMSC-EGFR) and dose rates, as well as their interaction, followed by post hoc multiple comparisons to evaluate differences between groups at each dose rate. Error bars represent standard deviation (SD). Statistical significance was considered when *p*-value < 0.05.

## 3. Results

### 3.1. EGFR mRNA Transfection Efficiency and Protein Expression

**Figure 1:**
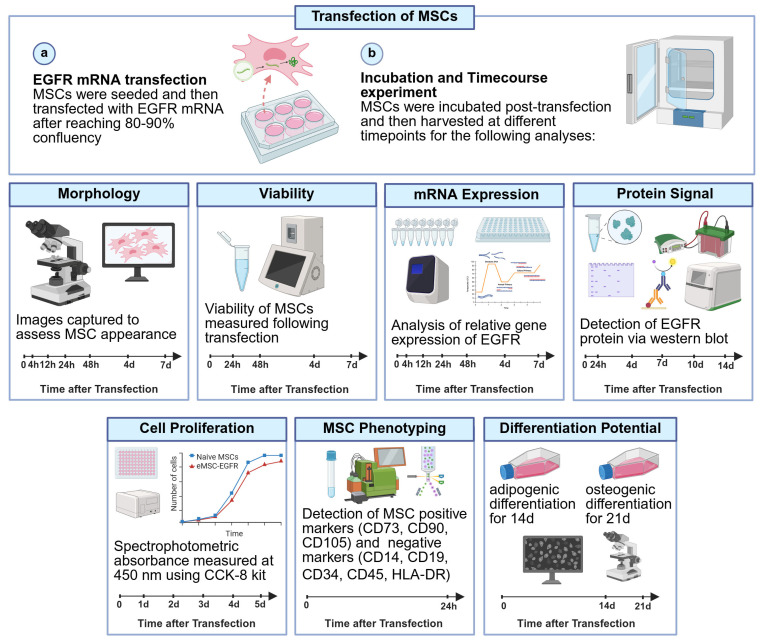
Schematic workflow of transfection of MSCs with EGFR. MSCs were transfected with EGFR mRNA and then the feasibility, efficiency, and stability of EGFR mRNA transfection into MSCs were analyzed via microscopy, qRT-PCR, immunoblotting, flow cytometry, cell proliferation assays and differentiation assays. (**a**) EGFR mRNA transfection (**b**) Incubation and time course experiment. Timeline added for each experiment with timepoints when data was collected. Created in BioRender. Ghani, F. (2025) https://BioRender.com/38y2614 (accessed on 10 July 2025).

To evaluate the feasibility, efficiency, and stability of EGFR mRNA transfection into MSCs, several analyses were performed (Figure 1).

**Figure 2:**
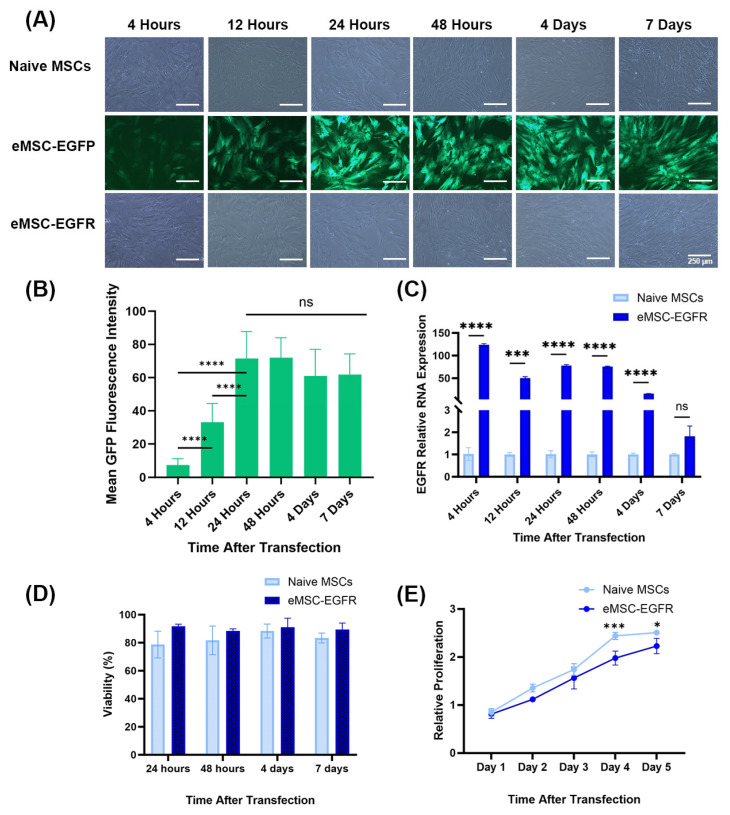
Evaluation of EGFR mRNA delivery feasibility and its stability within MSCs over time and expression of EGFR protein. (**A**) Representative images of naïve MSCs, eMSC-EGFP and eMSC-EGFR 4 h, 12 h, 24 h, 48 h, 4 d and 7 d after transfection. Scale bar: 250 µm. (**B**) Fluorescent intensities were measured in thirteen randomly selected cells and fields for background per image for each timepoint after transfection in eMSCs-EGFP. Mean background signal was subtracted from each mean fluorescence value. Quantification of green fluorescence intensity was performed using ImageJ Software. Statistical analysis was performed using unpaired with unequal variance, two-tailed Student’s *t*-test. (**C**) Quantification of EGFR RNA in eMSC-EGFR (*n* = 3). The EGFR mRNA level in naïve MSCs and eMSC-EGFR was detected after 4 h, 12 h, 24 h, 48 h, 4 days and 7 days of transfection. EGFR mRNA in eMSC-EGFR declined as time progressed. (**D**) Viability measures (*n* = 3). (**E**) Cell proliferation assay with data normalized to Day 1 at 450 nm. The proliferative capacity of MSCs was assessed by CCK-8 assay (*n* = 3). Data are presented as mean ± SD (standard deviation). * *p* < 0.05, *** *p* < 0.001, **** *p* < 0.0001 and ns indicates not significant.

A time course experiment was conducted using a 6-well plate culture system and monitoring expression of EGFP at 4 h, 12 h, 24 h, 48 h, 4 days, and 7 days post-transfection (Figure 2A–C). Most MSCs transfected with EGFP mRNA (eMSC-EGFP) showed EGFP expression throughout, indicating successful transfection. Microscope images for all samples show no evidence of toxicity with the concentration of Lipofectamine MessengerMAX (LMM) used in the experiment (Figure 2A). EGFP transfection reached an optimal level at 24 h and stayed the same afterwards, as indicated by the measurements of mean GFP fluorescence intensity (Figure 2B). Therefore, EGFP mRNA-engineered MSCs are viable and show a stable GFP expression with the majority of transfected MSCs showing GFP positivity.

As shown in Figure 2A, both eMSC-EGFP and eMSC-EGFR remained viable 7 days post-transfection. qRT-PCR was performed to assess the cellular uptake of EGFR mRNA transcripts in MSCs. EGFR mRNA was detected at high levels in eMSC-EGFR, which was over 120 times more than in naïve MSCs at 4 h post-transfection to more than 15 times at 4 days. However, there was no statistical difference in the EGFR relative RNA expression between naïve MSCs and eMSC-EGFR at day 7 (Figure 2C). Viability remained high throughout the time-course experiment with naïve MSCs and eMSC-EGFR showing similar viability measures (Figure 2D). Additionally, MSC proliferation was assessed using the CCK-8 assay. The results demonstrated that, while the relative proliferation of both naïve MSCs and eMSC-EGFR continued to increase as time progressed, the relative proliferation of eMSC-EGFR was significantly less than that of naïve MSCs on days 4 and 5 after transfection (Figure 2E).

**Figure 3:**
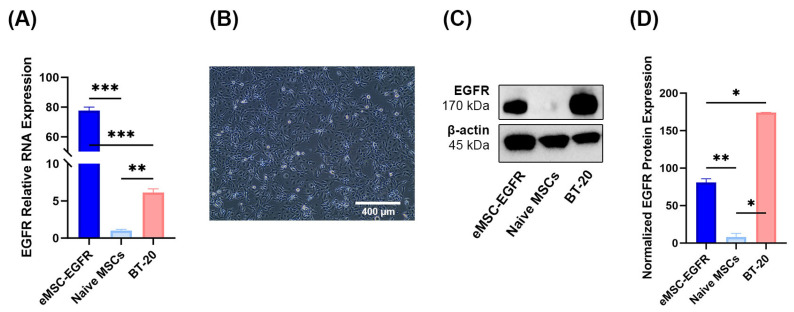
EGFR protein production and BT-20 cell line. (**A**) Relative EGFR RNA levels in eMSC-EGFR (*n* = 3), naïve MSCs (*n* = 3), and BT-20 cells (*n* = 3). Values are normalized to naïve MSCs. Cells were lysed for RNA isolation 24 h after transfection and following wash steps. (**B**) Representative images of BT-20 cells 24 h in culture. Scale bar: 400 µm (**C**,**D**) Cells were lysed for Western blot analysis 24 h after transfection. (**C**) Western blot analysis of EGFR protein levels in eMSC-EGFR, naïve MSCs and BT-20 cells. β-actin served as a loading control. Two independent experiments were performed. Representative Western blot illustrating protein expression levels. (**D**) Western blot protein band quantification with EGFR protein normalized to β-actin loading control using ImageJ Software (*n* = 3). Data are presented as mean ± SD. Statistical test used was ordinary one-way ANOVA with no pairing and matching with assumption of Gaussian distribution of residuals for EGFR relative RNA expression and EGFR immunoblotting analyses. * *p* < 0.05, ** *p* < 0.01, *** *p* < 0.001.

After monitoring the transfection of MSCs with GFP mRNA, EGFR engineered MSCs were compared with the known EGFR-expressing cell line BT-20 (Figure 3A). Prior to Western blot analysis of EGFR protein levels, BT-20 cells were in culture for 24 h (Figure 3B). The relative EGFR mRNA level in eMSC-EGFR was 77 times greater than in naïve MSCs and about 14 times greater than in BT-20 cells. Additionally, Western blot were performed to assess EGFR translation and protein expression (Figure 3C,D). Consistent with the qRT-PCR results, naïve MSC lysates produced almost undetectable levels of EGFR protein while both eMSC-EGFR and BT-20 cells strongly expressed EGFR. However, BT-20 cells exhibited approximately twice the protein band intensity compared to eMSC-EGFR, despite qRT-PCR results showing higher EGFR mRNA transcripts in eMSC-EGFR. This suggests that not all EGFR transcripts transfected into the MSCs were translated into protein. Altogether, these findings indicate that EGFR mRNA transfected MSCs are capable of both transcript uptake and protein translation.

### 3.2. Characterization of EGFR mRNA-Engineered MSCs

**Figure 4:**
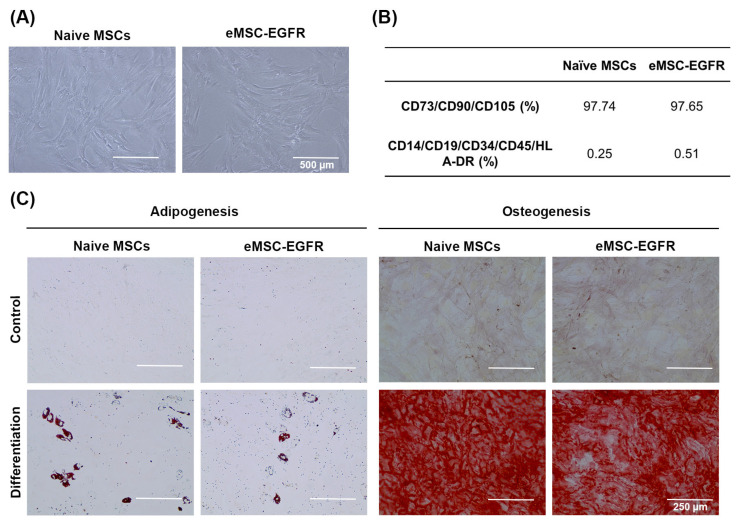
Identification of MSCs. (**A**) Representative images of naïve MSCs and eMSC-EGFR after 24 h of transfection. Scale bar: 500 µm. (**B**) Fluorescence-activated cell sorting (FACS) analysis of MSC surface marker expression after 24 h of transfection. MSC surface markers CD73, CD90 and CD105 were expressed in both naïve MSCs and eMSC-EGFR indicating the retention of the MSC phenotype. (**C**) Representative images of MSC differentiation assays. MSCs were cultured in adipogenic differentiation medium for 14 days, followed by staining with oil red-O. In parallel, MSCs were maintained in osteogenic differentiation medium for 21 days, followed by staining with alizarin red S. Scale bar: 250 µm.

To characterize eMSC-EGFR, their MSC identity was evaluated through morphological assessment, the flow cytometric analysis and functional differentiation assays toward adipogenic and osteogenic lineages. Following the phenotypic guidelines established by the International Society for Cell & Gene Therapy (ISCT) [15], eMSC-EGFR preserved their spindle or fibroblast-like adherent cell characteristics when compared to naïve MSCs (Figure 4A). Flow cytometry was used to confirm the expression of CD105, CD73 and CD90 and absence of CD45, CD34, CD19, CD14 and HLA-DR in MSCs (Figure 4B, Supplementary Figure S1). Additionally, eMSC-EGFR retained their ability to differentiate into adipogenic and osteogenic lineages (Figure 4C).

### 3.3. Cell Morphology and Proliferation Assessment of Irradiated MSCs

**Figure 5:**
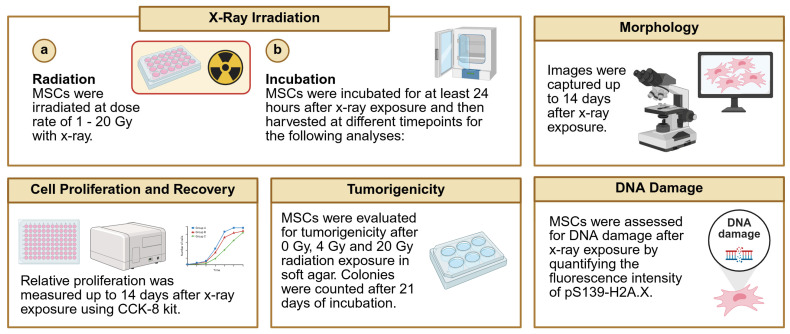
Schematic diagram of techniques used to assess MSCs following irradiation with X-ray. (**a**) Naïve MSCs and eMSC-EGFR were irradiated with X-ray at various dose rates and then (**b**) assessed for MSC characteristics including morphology, cell proliferation, tumorigenic potential and DNA damage. Created in BioRender. Ghani, F. (2025) https://BioRender.com/8ydwomy (accessed on 10 July 2025).

After confirming successful transfection of MSCs with EGFR and retaining their properties as MSCs, they were irradiated with a single dose of X-ray, using varying doses ranging from 1 to 20 Gy. Cells were then assessed after exposure (Figure 5).

**Figure 6:**
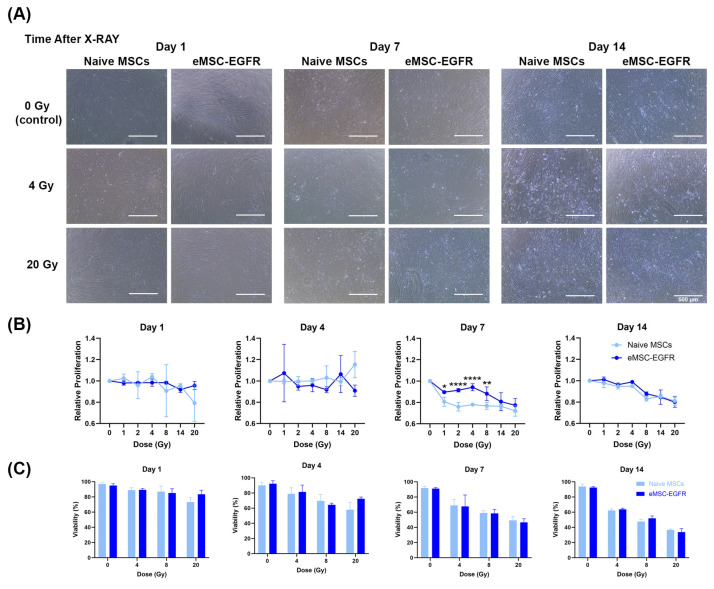
Naïve and engineered MSCs after single-dose X-ray exposure. (**A**) Representative images of morphology of naïve MSCs and eMSC-EGFR up to 14 days after X-ray exposure at dose rates 0–20 Gy. Scale bar: 500 µm. (**B**) Relative cell proliferation to control (0 Gy) and (**C**) cell viability after 14 days of X-ray exposure. All samples were analyzed in triplicate (*n* = 3). Data are presented as mean ± SD. * *p* < 0.05, ** *p* < 0.01, and **** *p* < 0.0001.

A cell proliferation assay was performed, and the morphology of cells exposed to different doses of X-ray was observed to assess the radiation responses of naïve MSCs and eMSC-EGFR. Images were captured at different timepoints following radiation exposure with greater evidence of floating cells (dead cells) as time progressed and with higher radiation dose (Figure 6A).

A cell proliferation assay was performed with an analysis assessing proliferation at each dose relative to proliferation at dose 0 Gy (no X-ray exposure). No difference in cell proliferation was observed between naïve MSCs and eMSC-EGFR at days 1, 4, and 14 following X-ray exposure. During days 1 and 4 after irradiation, there seem to be no differences between naïve MSCs and eMSC-EGFR at each dose tested, and similar relative proliferation across the different doses, indicating that there were no changes in total cell numbers between the samples at 0 Gy and irradiated ones. However, on day 14, although there are no differences between naïve MSCs and eMSC-EGFR, relative proliferation decreases as dose increases. This indicates that the total cell numbers were dropped as time progressed after irradiation, with a correlation to dose. Moreover, significant differences were observed between naïve MSCs and eMSC-EGFR on day 7 following X-ray exposure, with eMSC-EGFR demonstrating greater relative proliferation than naïve MSCs at dose 1–8 Gy, and no differences above 8 Gy, indicating that eMSC-EGFR showed radioresistance 7 days after irradiation at low doses. This observation had diminished when measured at day 14 (Figure 6B).

Cell viability was also measured and showed that dose rate has a strong effect on viability (*p* < 0.0001) at each timepoint; as dose increased, cell viability decreased (Figure 6C). Statistical analysis demonstrated that there was no overall difference between naïve MSCs and eMSC-EGFR when averaged across all dose rates. Statistical analysis also investigated a full model including the interaction effect (interaction between cell group and dose) and showed that the effect of dose rate was consistent across both naïve MSCs and eMSC-EGFR. Thus, the biological response is dose-dependent and not group-dependent.

### 3.4. Assessment of Irradiated MSCs for Tumorigenic Potential

**Figure 7:**
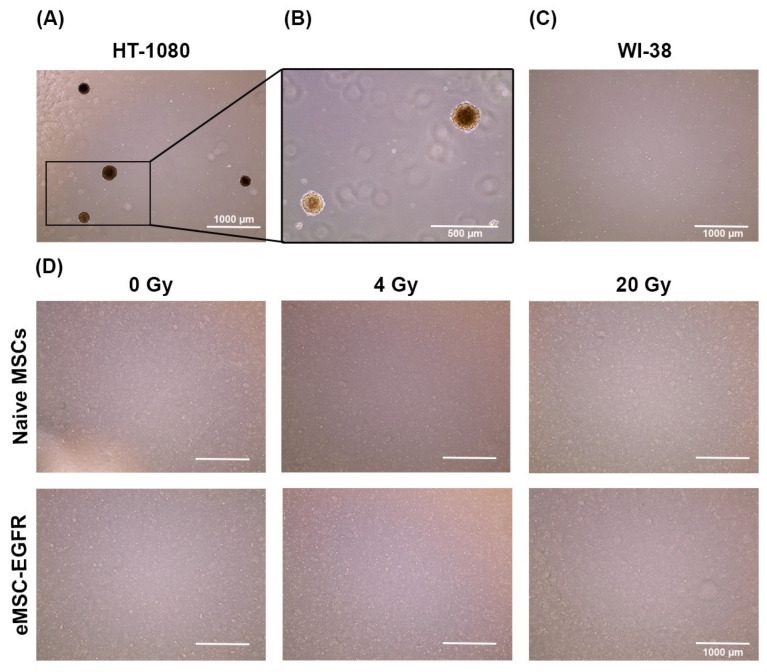
Tumorigenicity assay of naïve and engineered MSCs with EGFR after single-dose X-ray exposure. Cells were plated in soft agar to assess their ability to form colonies, indicative of tumorigenic potential. (**A**,**B**) 5000 cells/well of positive control cell line HT-1080 and (**C**) 67,000 cells/well of WI-38 negative control cell line were used as controls to validate the procedure. (**D**) 67,000 cells/well of naïve MSCs and eMSC-EGFR were cultured for three weeks to assess tumorigenic potential 24 h following no X-ray exposure (0 Gy), 4 Gy and 20 Gy (**A**,**C**,**D**) scale bar: 1000 µm (**B**) scale bar: 500 µm.

A tumorigenicity assay was performed to indicate whether there is any evidence of tumorigenic transformation in naïve MSCs and eMSC-EGFR after X-ray exposure. The presence of radiation may result in oncogenic mutations. Naïve and EGFR engineered MSCs showed no evidence of tumorigenic transformation after 4 Gy and 20 Gy X-ray exposure. Therefore, a tumorigenicity assay was used to evaluate naïve MSCs and eMSC-EGFR tumorigenic capacity after 4 Gy (low dose) and 20 Gy (high dose) exposure. HT-1080 served as a positive control cell line (Figure 7A–C) and showed the formation of colonies at both 5000 cells/well and 67,000 cells/well. WI-38 was used as a negative control cell line (Figure 7D) and showed no formation of colonies. Neither naïve MSCs nor eMSC-EGFR showed evidence of tumor formation at 4 Gy or 20 Gy even after 3 weeks of cell culture in the assay (Figure 7D).

### 3.5. Genomic Integrity Analysis of Irradiated MSCs

**Figure 8:**
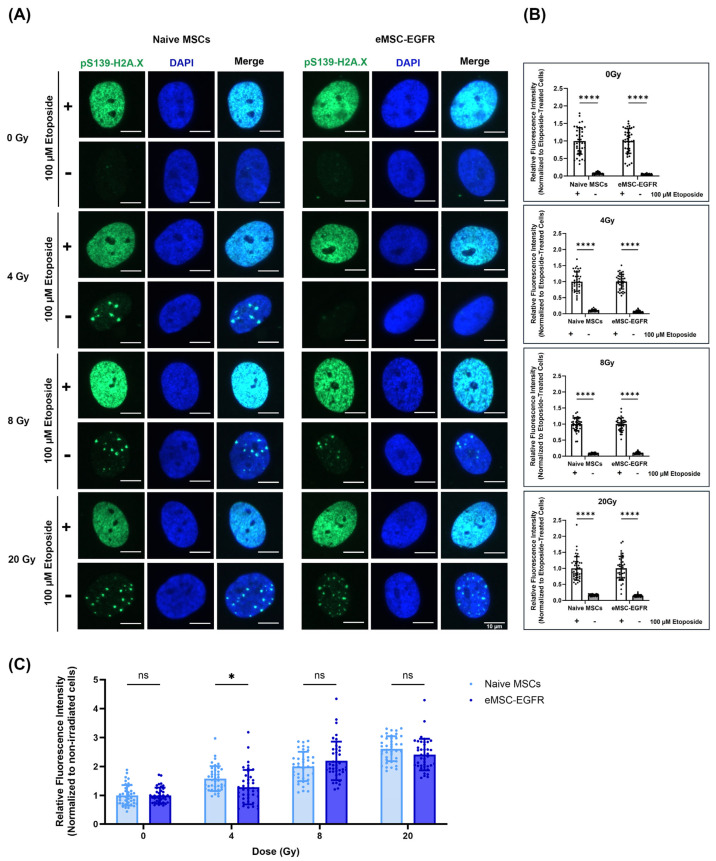
Evaluation of irradiated MSCs for DNA damage. (**A**) Representative images of bone marrow-derived mesenchymal stem cells (5000 cells/well) seeded to ibidi µ-Slide 8 Well demonstrating naïve MSCs and engineered MSCs overexpressing EGFR. Cells were exposed to X-ray as indicated, fixed, and subjected to immunofluorescence analysis to visualize pS139-H2A.X. In each group, cells were stimulated with 100 µM etoposide to serve as positive control of DNA damage. Scale bar: 10 µm (**B**,**C**) Quantification of the fluorescence intensity of pS139-H2A.X (*n* = 40 cells). Analysis was conducted using the ImageJ Software where the mean fluorescence intensity within each cell was measured followed by background subtraction, averaging, then normalization against the average measured from the non-irradiated cells or etoposide-treated cells. Forty cells were quantified for each sample. Data are presented as mean ± SD. (**B**) Fluorescence intensities were normalized to the etoposide-treated group for each experimental condition. **** *p* < 0.0001 was determined by two-way ANOVA, assessing the differences between the mean of the etoposide-treated cells and non-treated cells at each exposure group. (**C**) Fluorescence intensities at each X-ray exposure group and MSC condition were normalized to non-irradiated cells (0 Gy) within that same group. * *p* < 0.05 determined by parametric *t*-test (unpaired, two-tailed) with Welch’s correction while ns indicates not significant.

Irradiation induces DNA double-strand breaks (DSBs) in MSCs. γ-H2AX foci, a common marker for DSBs, typically form within minutes after irradiation and peak within 30–60 min. In most cell types, these foci diminish within 6–24 h as repair is completed. If no foci are observed at 24 h, this may indicate that repair mechanisms like non-homologous end joining (NHEJ) or homologous recombination (HR) have resolved the damage [16]. Therefore, DNA damage evaluation was performed by visualization and subsequent quantification of the phosphorylation of histone variant γH2A.X at serine 139, in the nuclear compartment of cells, while 4′,6-diamidino-2-phenylindole (DAPI) was utilized for fluorescent labeling of nuclear DNA (Figure 8). The phosphorylation of H2A.X at S139 is a hallmark of the early DNA damage signaling cascade triggered by DNA DSBs [17] and is therefore a commonly used marker for DNA damage [18].

We chose to evaluate DNA damage 24 h post-irradiation to determine the long-term effects of irradiation and the efficiency of DNA repair mechanisms (late response). So, immunofluorescence staining and microscopy were used to detect and measure γ-H2AX foci 24 h after X-ray exposure to assess the ability of naïve MSCs and eMSC-EGFR to efficiently repair DNA DSBs. Persistent DNA damage markers at this stage may indicate more severe or irreparable damage. Two hours prior to staining, a positive control was included in each group where cells were treated with 100 µM etoposide, an inducer of DNA DSBs.

In cells exposed to 0 Gy and 4 Gy, immunostaining showed the faint detection of pS.139-γH2A.X within cell nuclei (Figure 8A). The etoposide-treated cells for all groups showed high levels of DNA damage as indicated by strong detection of pS.139-γH2A.X, as expected (Figure 8A,B). At each X-ray exposure group, there was a significant difference between cells treated with etoposide and untreated cells regarding the nuclear content of pS.139-γH2A.X (Figure 8B).

When comparing the mean fluorescence intensity between naive MSCs and eMSC-EGFR when normalized to non-irradiated cells (0 Gy) at each exposure group, an increase in relative fluorescence intensity was observed as the X-ray dose increased (Figure 8C). Naive MSCs and eMSC-EGFR demonstrated approximately three times greater mean fluorescence intensity 24 h after exposure to 20 Gy compared to non-irradiated cells. There were no differences between the response of native MSCs and eMSC-EGFR 24 h after exposure to 8 Gy and 20 Gy. However, naive MSCs demonstrated a statistically greater relative fluorescence intensity, indicating higher DNA damage content, compared to eMSC-EGFR 24 h after exposure to 4 Gy.

## 4. Discussion

The purpose of this investigation was to explore the radioresistant potential of MSCs when engineered to overexpress EGFR for the protection of MSCs from deep space cosmic radiation. Many studies have shown the detrimental effects of ionizing radiation on Earth and in space on various parts of the human body, including the hematopoietic system [19]. Unfortunately, the hematopoietic system is one of the main target organs of irradiation injury [20]. Cosmic radiation in space is a serious health hazard and problem for travelers embarking on long-duration and deep space missions [21].

In this study, we first characterized the safety and feasibility of engineering MSCs to overexpress EGFR and then assessed their ability to protect MSCs from low-to-high dose radiation at several timepoints after exposure. MSCs were chosen because they are the predominant stem/stromal cell used in cell therapy clinical trials, and because they play a vital role in the production of many other cell types. Also, MSCs have been used in both basic research and experimental regenerative medicine applications since they can be isolated from various tissues and readily cultured in vitro.

### 4.1. Summary of Findings and Suggested Mechanisms

The first step towards establishing the successful and feasible transfection of MSCs with EGFR was ensuring that the phenotypic identity of MSCs was not affected by EGFR overexpression via mRNA transfection. Successful transfection was confirmed via the qPCR analysis which showed significantly greater EGFR relative RNA expression in eMSC-EGFR compared to naïve MSCs, and significantly greater EGFR protein production via Western blot (Figure 2 and Figure 3).

For almost two decades, mRNA transfection into mammalian cells has been studied [22,23]. Engineering stem cells using mRNA transfection is a method with many benefits. First, transfected mRNA is not required to enter the nucleus or integrate into the host genomic DNA which makes the risk of genetic modification highly unlikely [24]. Second, mRNA transfection via lipofectamine-based method does not provoke an immune response to viral antigens in vivo, unlike viral-based transfection [25]. Additionally, mature mRNA can be generated in a cell-free environment, reducing the risk of contamination from other cellular components in the final engineered product [26]. Various kinds of nucleotide modifications including 5′ UTR and poly A-tail give high translation efficiency and RNA stability. Also, as shown in the results, this method of mRNA transfection allows high transfection efficiency into MSCs, consistent with previous reports [22,23,27].

As shown in Figure 2, EGFR mRNA level in eMSC-EGFR was nearly 50–100 times greater than naïve MSCs, and eMSC-EGFR expressed around 75 times more EGFR proteins compared to naïve MSCs 1 day after EGFR mRNA transfection. Despite the significant elevation of EGFR expression, this did not seem to change the basic characteristics of MSC. Therefore, the overexpression of EGFR in MSCs in this study further develops the use of mRNA transfection as a method to enhance stem cells and engineer them to achieve certain biological or therapeutic outcomes such as protection from radiation-induced injury. Furthermore, the efficient mRNA transfection of multiple MSC donors using Lipofectamine-based method has been tested, optimized, and analyzed in our previous studies [27,28].

Radiation is a critical determinant of cellular response, with effects ranging from transient stress to irreversible damage depending on the magnitude and duration of exposure. In general, low-dose ionizing radiation (e.g., <1 Gy) may induce mild oxidative stress and DNA damage, often triggering repair mechanisms without significantly impairing proliferation. One study suggested that acute exposure to low-dose (0.1 Gy) radiation transiently affected functional characteristics of human bone marrow-derived MSCs with radiation-induced MSCs having recovered such that they were similar to non-irradiated cells [29]. Moderate to high doses, such as those used in this study (1–8 Gy), are known to cause DNA double-strand breaks, apoptosis, cell cycle arrest, and senescence, particularly in sensitive cell populations. While MSCs are relatively resistant compared to hematopoietic cells (HSCs), they still exhibit dose-dependent changes in proliferation, differentiation, and cytokine secretion following X-ray exposure [30].

In our study, both naïve MSCs and eMSC-EGFR exhibited a dose-dependent decrease in relative proliferation as time post-irradiation progressed. However, naïve MSCs showed a more pronounced reduction at day 7, following moderate-to-high radiation exposure 1–8 Gy, compared to eMSC-EGFR. This disparity may be explained by differences in EGFR expression and intrinsic proliferation rates. EGFR is a key regulator of cell survival and DNA repair, particularly following radiation-induced damage. Cells with higher EGFR expression, such as eMSC-EGFR, may activate more robust pro-survival and repair pathways, including the PI3K/AKT and DNA-PK-mediated mechanisms, thereby mitigating radiation-induced proliferation loss [31].

In contrast, naïve MSCs, which expressed significantly lower levels of EGFR, may lack sufficient signaling to counteract radiation stress, resulting in greater cell cycle arrest or senescence. Additionally, the natural proliferation rate is a critical factor in radiation susceptibility, where rapidly dividing cells are more vulnerable to radiation due to increased DNA replication stress and exposure during mitosis [32]. Naïve MSCs showed more rapid proliferation under baseline conditions in the absence of irradiation (Figure 2), and this could further compound its sensitivity to radiation. These findings suggest that both EGFR expression and proliferation kinetics contribute to the differential radiation response observed at day 7, highlighting the importance of intrinsic cellular properties in modulating radiobiological outcomes. Furthermore, our findings show that differences were only evident at moderate dose rates and not higher ones (>8 Gy), which has been observed previously [30].

Notably, no differences were observed on days 1, 4, or 14 post-irradiation, suggesting that the 7-day timepoint captures a critical window where intrinsic differences in radiation sensitivity and repair capacity become functionally apparent. This supports the notion that radiation effects are not only dose-dependent but also temporally dynamic, with delayed cellular responses revealing deeper biological distinctions between naïve and engineered MSCs. MSCs typically exhibit a natural doubling time of approximately 24 to 72 h under optimal conditions. Early timepoints (day 1 and 4) likely captured the immediate stress response, during which both cell types may have activated similar repair mechanisms or entered transient cell cycle arrest. By day 7, cells may have resumed proliferation, allowing intrinsic differences in repair efficiency, cell cycle regulation, or senescence induction to manifest as divergent growth rates. The absence of observed differences at day 14 suggests either a recovery or stabilization phase, where surviving cells have adapted to the radiation insult.

The emergence of differential proliferation between naïve MSCs and eMSC-EGFR at day 7 post-irradiation aligns with the literature showing that radiation-induced senescence responses in MSCs typically manifest between days 5 and 10. A study investigating the ionizing radiation-induced cellular senescence in human MSCs over a 10-day period showed that senescence-related changes became prominent around day 6 post-irradiation using X-ray dose of 4 Gy. Also, the 4 Gy dose was sufficient to induce the significant cytoskeletal reorganization and activation of senescence pathways including p53, p21, and p16 [33]. Thus, day 7 represents a biologically relevant window to detect the early but sustained effects of radiation on stem cell behavior, particularly in relation to their regenerative potential and susceptibility to long-term damage.

Since the most notable effects were observed at day 7 post-irradiation, this period likely reflects the culmination of early signaling cascades, DNA damage responses, and inflammatory processes initiated by irradiation. However, to fully delineate the temporal dynamics and potential delayed effects, it would be important to incorporate longer-term assays in future studies. Such analyses could uncover sustained or secondary responses that are not apparent at earlier timepoints, thereby providing a more comprehensive understanding of the biological impact of irradiation on naïve and engineered MSCs.

Because cells were exposed to ionizing radiation, there is still concern of tumorigenic transformation because of the correlation between ionizing radiation and oncogenic mutation [34,35,36]. Also, since EGFR is reported as an oncogene, the overexpression of EGFR in MSCs may raise concerns of oncogenic susceptibilities [37]. Wild-type EGFR overexpression transformed cells in vitro and induced tumorigenesis in vivo in transgenic mouse models, with tumor maintenance dependent on the continuous expression of EGFR [38]. Additionally, mutant EGFRs were shown to transform fibroblasts and lung epithelial cells, leading to anchorage-dependent growth and tumor formation in immunocompromised mice [39]. Elevated EGFR expression and increased EGFR copy number were prevalent in esophageal squamous cell carcinoma and contributed to malignant biological behaviors [40]. In cancer, EGFR overexpression is mostly due to EGFR gene amplification or mutations. Previous studies have shown an association between EGFR alterations and aggressive biological characteristics in different human cancers of epithelial origin [41,42,43,44,45,46,47,48,49,50,51,52].

Since EGFR is known to play a key role in the tumor microenvironment, a study investigated the effects of EGFR system on MSCs following stimulation with EGFR ligand. The findings revealed that microRNAs (miRNAs) modulated by EGFR in MSCs were potentially involved in cross-talk with breast cancer cells, highlighting the influence of the EGFR signaling system on MSCs [53]. Nonetheless, our findings show that EGFR overexpression in MSCs and exposure to X-rays does not cause tumorigenic formation within the timeframe investigated in this study.

Furthermore, DNA damage assessment demonstrated cells exposed to 0 Gy and 4 Gy showed faint detection of and quantification of pS.139-γH2A.X in cell nuclei. The most likely explanation is that the cells successfully repaired the DNA DSBs induced by the X-ray exposure within 24 h. However, when visualizing the cells exposed to X-ray dose above 8 Gy, a greater detection of pS.139-γH2A.X was found in the nuclei of cells, especially at 20 Gy. Greater DNA damage was experienced following X-ray exposure to high doses and some DNA DSBs remain unrepaired. Therefore, naïve MSCs and eMSC-EGFR possessed a greater capacity for repairing radiation-induced DNA damage following exposure to low- and moderate-dose rates than high-dose rates. The findings indicate that DNA damage caused by ionizing radiation in both naïve MSCs and eMSC-EGFR is dose-dependent, consistent with evidence reported in radiobiological studies [17,30].

Interestingly, the only difference in DNA damage response between naïve MSCs and eMSC-EGFR was detected 24 h after exposure to 4 Gy. Naïve MSCs demonstrated a statistically greater relative fluorescence intensity, indicating higher DNA damage content, compared to eMSC-EGFR. This implies that the engineered MSCs may have enhanced DNA repair mechanisms, protective modifications or resistance to moderate radiation-induced damage. This may also reflect a threshold in which repair mechanisms in naïve MSCs may begin to fail. These findings could model astronauts’ response to moderate acute responses. In our study, the 4 Gy X-ray dose is acute and delivered within seconds; however, astronauts in deep space missions receive chronic exposure spread over weeks or months. For instance, astronauts are exposed to 0.15–0.53 mGy/day in low Earth orbit missions on the ISS. In deep space missions, radiation exposure during lunar missions is 0.3–1.0 mGy/day [54] while during a Mars mission it would be 0.7–2.0 mGy/day [5,54,55]. The daily GCR dose equivalent on the lunar surface is approximately 2.6 times greater than the dose inside the ISS [56].

However, there are instances where astronauts do receive acute doses up to 1–2 Gy such as during solar particle events (SPEs) with the absorbed dose being 10.48 mGy/day [57]. In our study, the wide range of X-ray dose rates studied (1–20 Gy) allowed modeling both sub-lethal and lethal effects to help define thresholds for DNA damage, transformation, stem cell viability, differentiation potential and tumorigenicity. It ensures that the possible dose rates MSCs may be exposed to in space are covered. SPEs can exceed the annual GCR dose in a single event, especially on the lunar surface or during Mars interplanetary travel where natural shielding is minimal [54]. The results of this experiment may closely reflect acute dose delivery in space rather than prolonged chronic exposure. Furthermore, the acute, high-dose exposures tested here (up to 20 Gy) are far beyond realistic astronaut conditions and represent possible stress tests in relation to the chronic, low-dose exposures encountered in space.

The transfection of EGFR into MSCs has had a functional impact on how cells respond to DNA damage with several biological mechanisms potentially explaining this. For instance, EGFR overexpression can activate downstream signaling pathways that promote DNA repair, such as the PI3K/AKT pathway which enhances cell survival and activates DNA repair proteins [58,59], and MAPK/ERK pathway which can regulate cell cycle checkpoints and repair machinery [60]. EGFRs may translocate to the nucleus and interact with DNA-PK, a key player in non-homologous end joining (NHEJ). Additionally, EGFR activation has been shown to suppress TNF-α-induced apoptosis by phosphorylating TNFR1 and modulating downstream signaling. This suppression involves AKT activation, which is known to stabilize Bcl-2 family proteins. This may allow cells to survive longer and repair damage rather than undergoing cell death [61]. Moreover, the potential mechanisms and pathways proposed here remain to be fully elucidated and should be rigorously investigated in future research to substantiate their functional relevance.

Also, EGFR activation can alter the cell cycle, potentially causing cells to accumulate in phases (like G1 or G2) where DNA repair is more efficient, and reducing the accumulation of DNA damage markers at a given timepoint. The same study also found that EGFR signaling may reduce oxidative stress or enhance antioxidant responses, leading to less secondary DNA damage from reactive oxygen species (ROS) after radiation [62]. EGFR’s role in enhancing DNA repair and reducing ROS implies a potential reduction in γ-H2AX fluorescence intensity, as fewer unrepaired DSBs would be present at a given timepoint. One study showed that EGFR deletion in hematopoietic stem and progenitor cells led to increased DNA damage and impaired recovery after irradiation, and that EGF treatment (activating EGFR) enhanced DNA repair via DNA-PKcs in both murine and human HSCs [63]. Therefore, EGFR expression levels modulate cellular resilience to moderate radiation dose rates.

Furthermore, since our results showed differences in DNA damage between naïve MSCs and eMSC-EGFR at 4 Gy, this may reflect a threshold for repair where eMSC-EGFR. No differences in DNA damage between naïve MSCs and eMSC-EGFR were observed at 8 or 20 Gy. This may reflect a dose-dependent threshold beyond which cellular repair mechanisms become saturated [17]. At moderate doses like 4 Gy, EGFR-mediated DNA repair pathways, such as activation of DNA-PKcs involved in NHEJ, may still function effectively, allowing eMSC-EGFR to repair damage more efficiently than naïve MSCs. However, at higher doses, the extent of DNA damage likely overwhelms these repair systems in both cell types, leading to uniformly high levels of residual damage. Additionally, high-dose radiation may induce widespread apoptosis or necrosis, reducing the population of damaged cells and masking earlier differences [64]. The 24 h post-irradiation timepoint may also capture a phase where repair is still ongoing at 4 Gy but largely ineffective at higher doses, further contributing to the convergence in DNA damage levels. These findings suggest that EGFR-associated protection is most evident at moderate radiation doses, where repair capacity is challenged but not yet exceeded.

### 4.2. Implications for Deep Space Travel

To our knowledge, this is the first study to report the use of *EGFR* as a target gene for overexpression in MSCs specifically aimed at enhancing protection against simulated cosmic radiation. EGFR has been shown to display resistance against ionizing radiation in the following cancers: non-small-cell lung cancer [65,66,67,68], glioblastoma [69], breast cancer [70], head and neck squamous cell carcinoma [71], and colorectal cancer [72]. Understanding how EGFR contributes to radiation resistance in MSCs may offer valuable insights for space research, where radiation exposure poses significant risks to astronaut health and tissue regeneration. This type of research could specifically inform several strategies to support deep-space missions. For instance, pharmacological agents that modulate EGFR signaling might be used to enhance cellular resilience against cosmic radiation. MSCs engineered to express protective EGFR pathways could serve as regenerative therapies for radiation-induced tissue damage. Additionally, EGFR could be explored as a biomarker to assess individual susceptibility to radiation, enabling personalized countermeasures. These insights could also guide the development of biological shielding or synthetic biology systems designed to maintain tissue integrity during prolonged exposure to space radiation, ultimately improving astronaut health and mission sustainability.

Our study has set the foundation for the further exploration of other potential target radioprotective genes using multiple cell types as models for space radiation exposure. Our study shows that the overexpression of MSCs with a gene that has shown radioresistant properties may offer radioprotective effects. MSCs possess a baseline radioresistance, primarily attributed to their efficient DNA damage response mechanisms [73,74]. Also, their quiescent nature often residing in the G0 phase, and ability to engage in autophagy as a protective mechanism to clear damaged organelles and proteins contribute to their radioresistance [75]. Hence, it is important to figure out how to make them even more radioresistant or if their radioresistant pathways can be enhanced in preparation for manned space travel into deep space. This knowledge can also be beneficial in their potential use in regenerative therapies following radiation injury. Overall, we propose that eMSC-EGFR might be a promising start to enhancing MSCs and other stem cells to be protected from radiation. Further research is required to fully evaluate the effectiveness of eMSC-EGFR as a therapeutic option for space travelers, and whether the systemic overexpression of EGFR affects the expression of other genes and their downstream effects.

### 4.3. Limitations

While no tumorigenic effects were observed in our study, EGFR is an oncogene that may interact with other genes to influence cancer risk, as oncogenic transformation typically involves multiple genetic and environmental factors. Hence, more studies are needed to fully evaluate the effectiveness and safety of overexpressing EGFR in MSCs and other cell types for radioresistant properties. Moreover, the three-week tumorigenicity assessment window was relatively short, especially in the context of capturing delayed radiation-induced cellular responses like mitotic-linked cell death, genomic instability, or tumor formation. Although the tumorigenicity assay was suitable for the initial screening of X-ray exposure effects, a more robust evaluation including longer-term assays (8–16 weeks) are recommended in future studies.

Additionally, other sources of MSCs should be investigated for their ability to protect from radiation with EGFR overexpression. One study showed that adipose tissue-derived MSCs exhibit stronger radioresistance compared to umbilical cord-derived MSCs and gingival tissue-derived MSCs due to their ability to effectively repair irradiation-induced DNA damage, contributing to reduced apoptosis and the up-regulated expression of stemness-associated genes [30]. These findings enhance our understanding of the radiation-induced responses of MSCs and could inform improved strategies for irradiation-induced tissue damage.

While our study confirmed successful transfection and protein expression at 24 h post-transfection, we did not assess the duration or stability of expression beyond this timepoint. Future studies will aim to characterize the time-dependent expression profile, including long-term expression up to and beyond 7 days, to better understand the persistence and functional relevance of EGFR.

While X-ray exposure performed in the laboratory provides a convenient and controlled method for studying radiation-induced cellular responses, it does not fully consider the complexity of cosmic radiation where high-linear energy transfer (LET) radiation and various radiation species exist in deep space. Cosmic radiation is highly dynamic with various doses and exposures existing all at once. Therefore, follow-up studies using high-energy protons and heavy ions, such as ^56^Fe or ^28^Si, which are key components of galactic cosmic rays, are needed [21,76]. These particles trigger distinct biological effects, including clustered DNA damage, oxidative stress, and changes in stem cell function, which may differ markedly from the effects induced by X-rays [77]. An investigation of the differential effects of X-ray exposure and ^56^Fe on human MSCs showed that in vitro osteogenic differentiation processes were not impaired, but that G2/M cell cycle arrest and DNA replication and binding activities were impacted more intensely by 1 Gy ^56^Fe ions than by 1 Gy X-rays [78].

Future experiments using particle accelerators or space analog facilities like the NASA Space Radiation Laboratory will be essential to evaluate the combined impact of mRNA transfection and simulated space radiation on MSC viability, differentiation, and therapeutic potential. Also, it is worth investigating the radioprotection of EGFR in engineered MSCs when exposed to other radiation types like gamma rays and ultraviolet (UV).

Another limitation includes the fact that the microgravity environment in space may impose confounding effects and change how cells respond to cosmic radiation. Nonetheless, very little is known about the effects of an actual cosmic radiation environment beyond the low Earth orbit on biological systems since humans have yet to embark on such missions.

## 5. Conclusions

In summary, the mRNA transfection of MSCs with EGFR is an effective way to enhance stem cells’ radioprotective abilities, especially at moderate X-ray doses. Our findings indicate that overexpressing EGFR preserves MSC identity and may increase their resistance to radiation, a promising step toward protecting cells during deep space travel. Continued research is needed to fully realize the potential of engineered MSCs as a countermeasure against cosmic radiation.

## Data Availability

The data of this study are available from the corresponding author upon reasonable request.

## Supplementary Materials

The following supporting information can be downloaded at https://www.mdpi.com/article/10.3390/cells14211719/s1, Figure S1: Flow cytometry plots that illustrate the gating strategy used for MSC characterization. Initial gating was performed to exclude debris and doublets based on forward and side scatter properties (FSC/SSC). Single cells were identified followed by sequential gating to identify specific cell subsets based on marker expression (CD73, CD90, CD105). Each panel shows the progression of gating steps, with percentages indicating the proportion of cells within each gate. (A) Naïve MSCs and (B) eMSC-EGFR.
