# Supplementary material for: EGFR mRNA-Engineered Mesenchymal Stem Cells (MSCs) Demonstrate Radioresistance to Moderate Dose of Simulated Cosmic Radiation
Source: Cells. 2025 Nov 1;14(21):1719. doi: 10.3390/cells14211719 (PMC12610173; doi:10.3390/cells14211719)
Supplement: Supplementary file 1 [file cells-14-01719-s001.zip › cells-3863123-supplementary.pdf]

Supplementary Materials

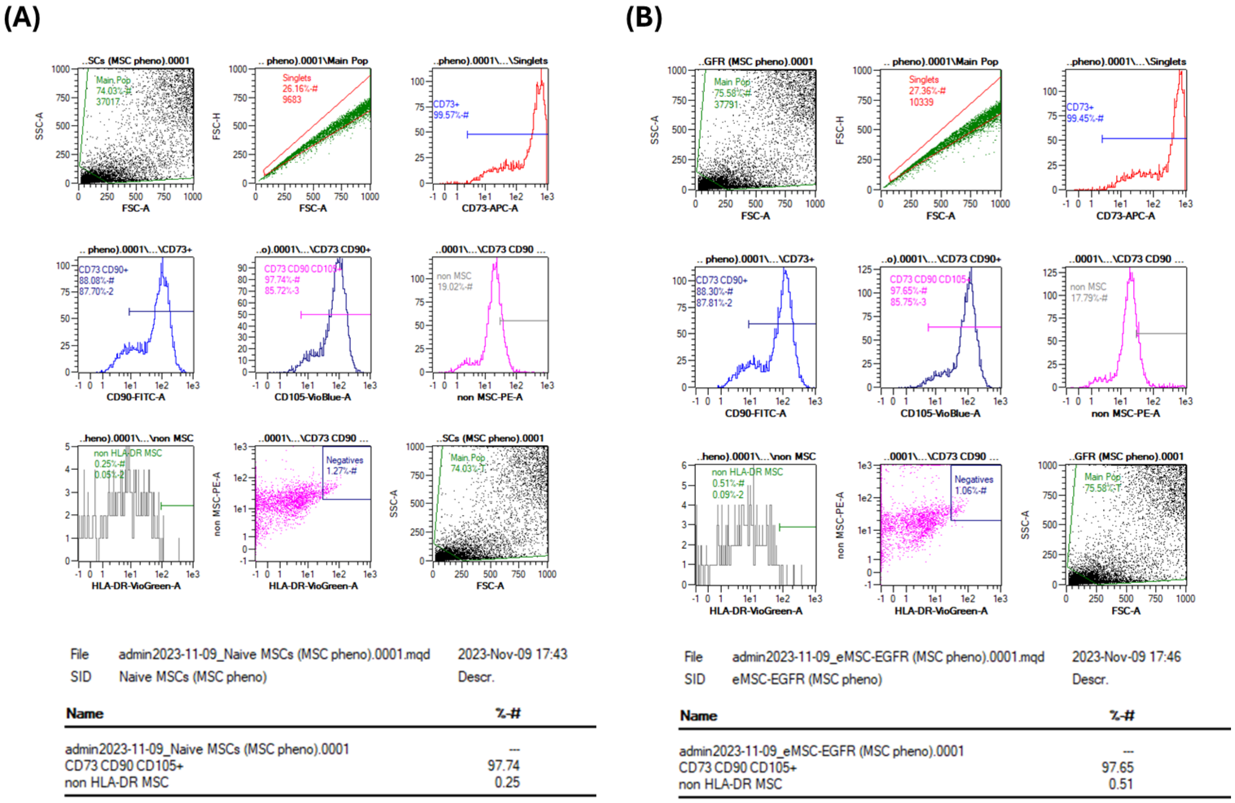

Figure S1. Flow cytometry plots that illustrate the gating strategy used for MSC characterization. Initial gating was performed to exclude debris and doublets based on forward and side scatter properties (FSC/SSC). Single cells were identified followed by sequential gating to identify specific cell subsets based on marker expression (CD73, CD90, CD105). Each panel shows the progression of gating steps, with percentages indicating the proportion of cells within each gate. **(A)** Naïve MSCs and **(B)** eMSC-EGFR.
